# Removal of Mixed Noise in Hyperspectral Images Based on Subspace Representation and Nonlocal Low-Rank Tensor Decomposition

**DOI:** 10.3390/s24020327

**Published:** 2024-01-05

**Authors:** Chun He, Youhua Wei, Ke Guo, Hongwei Han

**Affiliations:** 1 College of Geophysics, Chengdu University of Technology, Chengdu 610059, China; murphy222@cwnu.edu.cn; 2 Geomathematics Key Laboratory of Sichuan Province, Chengdu University of Technology, Chengdu 610059, China; guoke@cdut.edu.cn; 3 Education and Information Technology Center, China West Normal University, Nanchong 637009, China; 4 College of Mathematics and Physics, Chengdu University of Technology, Chengdu 610059, China; 5 Engineering and Technical College, Chengdu University of Technology, Leshan 614000, China; hhw_666666@163.com

**Keywords:** hyperspectral image, mixed noise removal, subspace representation, low-rank tensor decomposition, global spectral low-rank, spatial nonlocal self-similarity

## Abstract

Hyperspectral images (HSIs) contain abundant spectral and spatial structural information, but they are inevitably contaminated by a variety of noises during data reception and transmission, leading to image quality degradation and subsequent application hindrance. Hence, removing mixed noise from hyperspectral images is an important step in improving the performance of subsequent image processing. It is a well-established fact that the data information of hyperspectral images can be effectively represented by a global spectral low-rank subspace due to the high redundancy and correlation (RAC) in the spatial and spectral domains. Taking advantage of this property, a new algorithm based on subspace representation and nonlocal low-rank tensor decomposition is proposed to filter the mixed noise of hyperspectral images. The algorithm first obtains the subspace representation of the hyperspectral image by utilizing the spectral low-rank property and obtains the orthogonal basis and representation coefficient image (RCI). Then, the representation coefficient image is grouped and denoised using tensor decomposition and wavelet decomposition, respectively, according to the spatial nonlocal self-similarity. Afterward, the orthogonal basis and denoised representation coefficient image are optimized using the alternating direction method of multipliers (ADMM). Finally, iterative regularization is used to update the image to obtain the final denoised hyperspectral image. Experiments on both simulated and real datasets demonstrate that the algorithm proposed in this paper is superior to related mainstream methods in both quantitative metrics and intuitive vision. Because it is denoising for image subspace, the time complexity is greatly reduced and is lower than related denoising algorithms in terms of computational cost.

## 1. Introduction

Hyperspectral images (HSIs) contain vast amount of spectral information and spatial structure information due to the unique imaging mechanism and are widely used in various applications [1,2], such as object recognition [3,4], forest monitoring [5], earth observation [6], environmental protection [7], natural disaster monitoring [8], and military security [9], etc. However, HSIs are unavoidably corrupted by different types of noise during acquisition due to the presence of random errors, dark current, and thermal electronics [10,11], which greatly reduces the performance of subsequent processing for HSIs, including classification [12,13], unmixing [14,15,16], and target detection [17,18,19], etc. For the above reasons, removing noise from HSIs is a very imperative step before subsequent image processing [20].

So far, quite a few HSI denoising approaches have been put forward that have achieved certain denoising effects. Traditional denoising methods regard each band of HSI as an independent grayscale image and denoise each band image individually by using the band-by-band denoising technique. These approaches ignore the redundancy and correlation (RAC) among bands and fail to utilize the spectral and spatial structure information within HSI effectively; therefore, the overall denoising effect is not satisfactory. Typical traditional methods include BM3D [21], K-SVD [22], and WNNM [23].

To address the shortcomings of traditional denoising methods, many approaches utilizing spatial and spectral information have been put forward in recent years, including principal component analysis (PCA) [24], nonnegative matrix decomposition [25,26], sparse representations (SR) [27,28,29], and low-rank learning [30,31], etc. In reference [32], the authors proposed a low-rank nonlocal (LRSNL) method, which takes both spectral and spatial information into account and can remove mixed noise effectively. However, the method converts the HSI cube into a 2-D image for processing, resulting in incomplete spatial structure information obtained through the nonlocal method; therefore, there is still room for improvement. Reference [33] proposed a low-rank matrix recovery (LRMR) method, which considers a clean HSI as a low-rank matrix, allowing the mixed noise in HSI to be effectively removed by using the low-rank property. However, the LRMR method only considers the spectral correlation and ignores the spatial correlation. A total variation regularized low-rank matrix factorization (LRTV) method was proposed in the reference [34], where the total variation regularization term obtains spatial information of pixels but fails to utilize spectral information effectively and only has a good denoising effect on low-intensity noise; therefore, there is also much room for improvement. The LRTDTV method [35] uses tucker decomposition based on LRTV, which makes full use of the spectral correlation information. Its denoising effect is significantly better than that of LRTV, but it neglects the protection of HSI edge information. Therefore, the algorithm still needs further improvement.

Because an HSI is composed of a set of 2-D images that can be viewed as a 3-D tensor that naturally retains the spatial structure information of HSI, scholars have proposed many denoising methods based on low-rank tensor decomposition [36,37,38]. Most of these methods use a tensor kernel norm defined based on tensor singular value decomposition (t-SVD) for noise reduction. Using this property, a tensor robust principal component analysis (TRPCA) method was proposed [39], which utilizes both low-rank and sparsity of data and uses tensor kernel norm as a low-rank constraint so that Gaussian noise and sparse noise in HSI can be effectively removed. However, this approach utilizes the traditional $l_{1}$ -norm to constrain sparse terms, making the algorithm less accurate. Reference [40] proposed a low-rank tensor factorization method for spatial–spectral total variation regularization (SSTV-LRTF). The total variation (TV) regularization term maintains spatial piecewise smoothness while removing Gaussian noise; meanwhile, low-rank property can obtain the correlation of adjacent bands. Overall, the denoising approaches via low-rank tensor decomposition have achieved relatively effective denoising performance by fully preserving useful spatial structure information.

It is well known that HSI has much RAC in both the spatial domain and spectral domain and exists in a global spectral low-rank subspace; therefore, spectral low-rank constraints can be used for dimension reduction, as well as denoising. At present, there are relatively few subspace-based denoising algorithms for HSI. Reference [41] proposed an algorithm, FastHyDe, which obtains subspace representation of HSI through projection and then removes noise on subspace. On this basis, the authors also proposed a GLF algorithm [42]. The algorithm performs low-rank tensor decomposition for similar nonlocal 3-D image patches in subspace to remove noise and achieves good results. The FastHyMix algorithm [43] is a parameter-free mixed noise removal method for HSI, which uses a Gaussian mixture model to characterize the complex distribution of mixed noise and exploits the two main properties of hyperspectral data, namely, the low-rank in the spectral domain and the high correlation in the spatial domain. Because this method can extract the deep image before the neural denoising network, it runs much faster than other denoising algorithms. He et al. [44] utilized the spectral information and spatial structure of HSI to denoise the image on the subspace and then optimized the denoising results through iterations, achieving ideal denoising performance. Reference [45] proposed that the spectral features of HSI are located in a subspace and exploited low-rank decomposition to solve spatial nonlocal self-similarity. Meanwhile, the augmented Lagrangian method (ALM) was used to optimize the denoising model, and good denoising effects were also obtained. A tensor subspace low-rank learning method with nonlocal prior TSLRLN was proposed in reference [46]. In this method, the original noisy HSI tensor is projected into a low-dimensional subspace, and then the orthogonal tensor basis and tensor coefficients of the subspace are learned alternatively. The method also achieves positive denoising results due to fully utilizing the low-rank property of spatial and spectral of the HSI tensor. For the time being, combining subspace with low-rank tensor decomposition for HSI denoising is a research hotspot, and although some achievements have been made, there is still a long way to go.

Because of this, a new mixed noise-removing algorithm for HSI via subspace and nonlocal low-rank tensor decomposition is put forward in this paper. Firstly, the algorithm obtains the subspace representation (i.e., orthogonal basis and RCI) of HSI by utilizing RAC among the bands. Then, similar 3-D image patches in the RCI are grouped to form 3-D tensors, which are then denoised using low-rank tensor decomposition and the improved wavelet threshold method according to the nonlocal self-similarity of HSI. ADMM is used to optimize the orthogonal basis and RCI alternately to obtain the denoised HSI. After that, the denoised HSI is regularized iteratively, and the final denoised HSI is obtained through iterations. We name the algorithm SNLTAI, and its contributions are listed as follows:

1. The subspace representation is realized by using spectral low-rank constraint, which is obtained by projecting noisy HSI onto the orthogonal basis. The algorithm denoises the RCI obtained from the subspace representation instead of denoising all band images, which greatly reduces the complexity and saves the running time of the algorithm.
2. The low-rank tensor decomposition and the improved wavelet threshold denoising algorithm are successively used to denoise 3-D tensors constructed using nonlocal similar 3-D image patches in RCI. The improved wavelet threshold denoising algorithm results in a more thorough denoising of RCI. The orthogonal basis and RCI are updated using the ADMM algorithm to improve the denoising performance. The denoised HSI is iteratively regularized, so the final denoised HSI is obtained through iterations.
3. At present, many HSI denoising algorithms have considered mixed noise, but the ability to remove heavy mixed noise is extremely limited, and some algorithms are entirely unable to do so. The denoising model constructed using this algorithm has an excellent ability to remove strong mixed noise from HSI, which helps in recovering the image disturbed by strong noise so that the image can be efficiently processed accordingly for subsequent processing.

The sections are arranged as follows: Section 2 introduces the proposed HSI denoising model; Section 3 illustrates the specific steps of this algorithm; the experimental results of different algorithms for the simulated dataset and the real dataset, as well as the analysis of the corresponding parameters, are presented in Section 4; and Section 5 summarizes the paper.

## 2. Denoising Model for HSI

Suppose that $y\in R^{r\times c\times b}$ represents the observed noisy HSI cube, where r×c denotes the size of each band and b is the number of spectral bands. During processing, every band image is stretched into a row vector, then the HSI cube is reconstructed as a 2-D matrix $Y\in R^{b\times h}\;$ ( h=r×c ). The noise model for HSI can be formulated as:

(1) Y=X+S+N,

where Y and $X\in R^{b\times h}$ represent noisy HSI and clean HSI, respectively, and S denotes sparse noise, which contains impulse noise, stripe noise, and deadlines. N denotes Gaussian noise.

Because the spectrum of the noisy HSI Y contains a large amount of RAC, a reasonable assumption is that the valid information of the clean HSI X exists in a spectral low-dimensional subspace that can be approximately estimated using the noisy HSI Y . The estimation formula is denoted as X=EZ . E is the orthogonal basis of the subspace, with $E\in R^{b\times k}$ and k≪b , and k is the dimension of the subspace. $Z\in R^{k\times h}$ represents the representation coefficient of the subspace. Therefore, the noise model can be reformulated as:

(2) Y=EZ+S+N

According to the noise model, the denoising model for HSI can be formulated as:

(3) $$\underset{\;\;\;\;\;\;\;\;\;\;\;\;\;\;\;E,Z,S}{\left\{\hat{E},\hat{Z},\hat{S}\right\}=\arg}min\frac{1}{2}{\left||Y-EZ-S|\right|}_{F}^{2}+\lambda_{1}\phi_{1}\left(Z\right)+\lambda_{2}\phi_{2}\left(S\right),$$

where the first term on the right side of Formula (3) represents the fidelity term of the data, which is considered to be zero-mean Gaussian-independent and identically distributed noise (other covariance matrices can be dealt with using the methods in reference [47]), and ${\left||\cdot |\right|}_{F}^{2}$ represents the Frobenius norm. The second term is a regularization term related to the representation coefficient, which requires a low-rank constraint and can be processed with the nuclear norm [48]. The third term is utilized as the regularization term of sparse noise and can be suppressed by using the $l_{1}$ -norm of the matrix. $l_{1}$ -norm is also known as the Lasso regularization. The parameters $\lambda_{1}$ , $\lambda_{2}\geq 0$ are scale coefficients, which are used to balance the overall effect of denoising.

Because E is an orthogonal basis, we have $E^{T}E=I_{k}$ , and $I_{k}$ is the k -order identity matrix. Reference [49] highlighted that an orthogonal basis can reduce the complexity of the denoising model and accelerate the convergence of the algorithm.

After adding the constraint term of the orthogonal basis, the denoising model (3) can be rewritten as:

(4) $$\underset{\;\;\;\;\;\;\;\;\;\;\;\;\;\;\;E,Z,S}{\left\{\hat{E},\hat{Z},\hat{S}\right\}=\arg}min\frac{1}{2}{\left||Y-EZ-S|\right|}_{F}^{2}+\lambda_{1}{\left||Z|\right|}_{*}+\lambda_{2}{\left||S|\right|}_{1},\;\;\;\;s.t.\;\;E^{T}E=I_{k},$$

where ${\left||Z|\right|}_{*}$ represents the nuclear norm of Z , and $S_{1}$ denotes the $l_{1}$ -norm of S . The key to solving model (4) lies in the continuous optimization of the orthogonal basis E and the representation coefficient Z , which is described in detail in our algorithm later.

## 3. Proposed Denoising Method for HSI

In this part, we provide a detailed description of the proposed algorithm. The algorithm includes four steps: (1) spectral low-rank; (2) spatial nonlocal self-similarity; (3) updating orthogonal basis and representation coefficient; and (4) iterative regularization.

**Figure 1:**
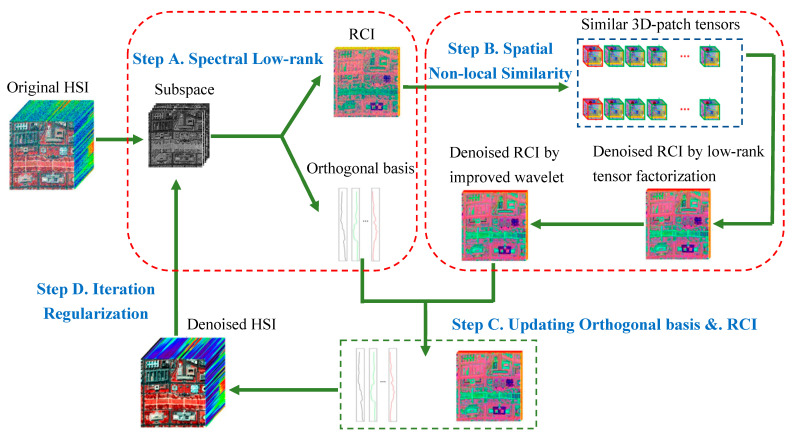
Flowchart of the proposed algorithm.

The overview of the proposed algorithm is shown in Figure 1.

### 3.1. Spectral Low-Rank

Due to the high RAC among the spectral bands, the valid information of a clean HSI exists in a low-dimensional subspace, which we assume to be $S_{k}$ , where k is the dimension of the subspace and k≪b . Hysime [50] is an efficient algorithm for estimating the dimension of the subspace of HSI based on the principle of least squares, which can be used to estimate the correlation matrix between the signal and the noise and thus simultaneously compute the dimension k of the subspace and the orthogonal basis $E_{all}$ of the signal space. The first k -column vectors of $E_{all}$ form the orthogonal basis E of the subspace $S_{k}$ .

Because there are hundreds of spectral bands in HSI, denoising for all bands is often time-consuming and does not yield good results. Therefore, performing low-rank spectral denoising on subspace can save a significant amount of processing time and achieve better denoising results. The orthogonal basis E of subspace $S_{k}$ can be projected on the observed HSI Y to obtain the representation coefficient Z of subspace $S_{k}$ , that is, $Z=E^{T}Y$ , and $Z\in R^{k\times h}$ . Each row element of the matrix Z is reshaped into a matrix of size r×c , which we name the eigen-image. All the eigen-images form the representation coefficients image (RCI) of the subspace. In other words, an RCI consists of k eigen-images. Like all natural images, each eigen-image has nonlocal self-similarity, and there exists a high correlation among the eigen-images [41,42]. In addition, if the noisy HSI follows Gaussian distribution with zero-mean and variance $\sigma^{2}$ , then the RCI still follows the same distribution [44]. Therefore, denoising for HSI can be transformed into denoising for its RCI.

Because the model involves iterative regularization, each round of the noisy image Y is obtained from the previous round of iterative processing, and the orthogonal basis E , representation coefficients Z , and sparse noise S are generated through the noisy image Y . The denoising model with the addition of iterative regularization is (subscript i denotes the i-th iteration):

(5) $$\begin{array}{c} \underset{\;\;\;\;\;\;\;\;\;\;\;\;\;\;\;\;\;E_{i},Z_{i},S_{i}}{\left\{{\hat{E}}_{i},{\hat{Z}}_{i},{\hat{S}}_{i}\right\}=arg\;min}\frac{1}{2}{\left||E_{i}^{T}Y_{i}-Z_{i}-E_{i}^{T}S_{i}|\right|}_{F}^{2}+\lambda_{1}{\left||Z_{i}|\right|}_{*}+\\ \lambda_{2}||S_{i}{\left|\right|}_{1},\;\;\;s.t.\;\;E^{T}E=I_{k} \end{array}$$

### 3.2. Spatial Nonlocal Self-Similarity

The processing in this stage includes the following three steps:

(1) Grouping similar 3-D image patches for RCI to form 3-D tensors;
(2) Performing low-rank tensor decomposition to denoise the 3-D tensor;
(3) The improved wavelet threshold method is conducted on the denoised 3-D tensor.

1. Grouping similar 3-D image patches to form 3-D tensors

In order to obtain the groups composed of similar 3-D image patches from RCI, the mean value matrix $RCI_{mean}$ needs to be calculated, and then all 2-D reference patches and 2-D image patches in $RCI_{mean}$ are obtained according to the step sizes of 5 and 1, respectively. Each 2-D reference patch corresponds to multiple overlapping 2-D image patches. Image patches need to overlap to avoid artifacts in the later image recovery process [45].

To obtain the n 2-D image patches that are most similar to the 2-D reference patch, instead of using Euclidean distance directly, we use the inner product of the improved Gaussian kernel function and Euclidean distance to measure the similarity of two image patches [51]. Euclidean distance sets the same weight for each position of the image patch, failing to highlight the role of the central pixel point of the image patch. However, the closer the distance between a certain point and the center point is, the greater the impact will be; otherwise, the lesser the impact will be [52]. Compared with the original Gaussian kernel function, the improved Gaussian kernel function can better highlight the weight of the center pixel point in an image patch. The experimental results demonstrate that the use of the improved distance provides a more accurate measure of similarity between image patches than the Euclidean distance and hence, better denoising results.

The original Gaussian kernel function formula is as follows:

(6) $$G\left(a,b\right)=\frac{1}{2\pi {\sigma_{0}}^{2}}exp^{-\frac{a^{2}+b^{2}}{2{\sigma_{0}}^{2}}},\left(-r\leq a,b\leq r\right),$$

where $\sigma_{0}$ is the variance of the Gaussian kernel function and r is the radius of the Gaussian kernel. [a, b] is the index of the row and column in the Gaussian kernel, and the index of the central pixel is [0,0].

The improved Gaussian kernel function formula is as follows:

(7) $$G\left(a,b\right)=\sum_{t=1}^{r}\frac{1}{2\pi {\sigma_{0}}^{2}}exp\left(-\frac{a^{2}+b^{2}}{2{\sigma_{0}}^{2}}\right),\left(-t\leq a,b\leq t\right),$$

Formula (7) accumulates Gaussian kernels with different radii based on the original Gaussian kernel function, with the radii ranging from 1 to r . The number of accumulations is also r , and the size of the final Gaussian kernel matrix is (2×r+1)×(2×r+1) .

After obtaining the n 2-D image patches that are most similar to each 2-D reference patch in $RCI_{mean}$ , we can obtain the n 3-D image patches that are most similar to each 3-D reference patch from the corresponding position in RCI. The above operation of extracting similar 3-D image patches from RCI is denoted by an operator G . The 3-D reference patch with index- ind is denoted by $R_{ind}\in R^{s\times s\times k}$ where s×s denotes the size of the reference patch, and k denotes the number of eigen-images. Then $G_{ind}Z\in R^{s^{2}\times k\times n}$ denotes the 3-D tensor formed by the n similar 3-D image patches corresponding to the 3-D reference patch $R_{ind}$ . We hope to obtain a clean 3-D tensor $C_{ind}$ by estimating the noisy 3-D tensor $G_{ind}Z$ . To this end, we built the following model:

(8) $$\underset{\;\;\;\;\;\;\;\;\;\;\;\;\;\;\;C_{ind}}{{\hat{C}}_{ind}=arg}min\sum_{ind}\left(\frac{1}{\sigma_{ind}^{2}}{\left||G_{ind}Z-C_{ind}|\right|}_{F}^{2}+\varphi ||C_{ind}{\left|\right|}_{*}\right),$$

where $\sigma_{ind}^{2}$ represents the noise variance of the noisy 3-D tensor $G_{ind}Z$ , which is used to normalize the fidelity term of the Frobenius norm, and $\varphi ||C_{ind}{\left|\right|}_{*}$ is the regularization to compute the nuclear norm of the tensor $C_{ind}$ , which is used to constrain the low rank of the clean tensor.

From the analysis above, the denoising model for HSI based on subspace and nonlocal low-rank tensor decomposition proposed in this paper is as follows:


(9) $$\begin{array}{c} \underset{\;\;\;\;\;\;\;\;\;\;\;\;\;\;\;\;\;\;\;\;\;\;E_{i},Z_{i},S_{i,}{\hat{C}}_{ind}}{\left\{{\hat{E}}_{i},{\hat{Z}}_{i},{\hat{S}}_{i},{\hat{C}}_{ind}\right\}=arg\;min}\frac{1}{2}{\left||E_{i}^{T}Y_{i}-Z_{i}-E_{i}^{T}S_{i}|\right|}_{F}^{2}+\lambda_{1}\underset{ind}{\sum }\left(\frac{1}{\sigma_{ind}^{2}}{\left||G_{ind}Z_{i}-C_{ind}|\right|}_{F}^{2}+\varphi ||C_{ind}{\left|\right|}_{*}\right)+\\ \lambda_{2}||S_{i}{\left|\right|}_{1},\;\;\;\;s.t.\;\;E^{T}E=I_{k}, \end{array}$$

2. Denoising the 3-D tensor using nonlocal low-rank tensor decomposition

The solution of model (9) is performed in the following way. The n 3-D image patches corresponding to each 3-D reference patch in RCI are operated as follows:

(1) Consider each 3-D image patch as a stack of k 2-D patches, expand each of these k 2-D patches into a column according to their order in RCI, and put these columns sequentially in a column to form a column vector. Then there are s×s×k elements in the column vector. All the n 3-D image patches are put in their respective column vectors in the same way to form a 2-D matrix, so the size of the matrix is (s×s×k)×n . Let t1 denote this matrix, i.e., $t1\in R^{\left(s\times s\times k\right)\times n}$ .

(2) Compute the mean value of each row of the matrix t1 so that there are a total of s×s×k values, and then expand the column vector with size s×s×k to n columns. Let t2 denote this matrix, i.e., $t2\in R^{\left(s\times s\times k\right)\times n}$ .

(3) Compute t=t1−t2 , and use the WNNM algorithm [23] to denoise the matrix t . After processing, each 3-D image patch needs to be placed at the corresponding position in RCI according to its index to realize the accumulation of element values, denoted as: $\underset{ind}{\sum }V_{ind}$ . Also, the number (weight) of 3-D image patches placed at each position needs to be accumulated, denoted as: $\underset{ind}{\sum }W_{ind}$ . Then $\underset{ind}{\sum }V_{ind}/\underset{ind}{\sum }W_{ind}$ is the denoised RCI.

It should be noted that the noise level of RCI should be estimated in advance during processing. It has been indicated that the noise level $\sigma_{RCI}^{2}$ of RCI is the same as that of the noisy HSI, so the noise level of the corresponding RCI can be obtained by estimating the noise level $\sigma_{i}^{2}$ of the current noisy HSI during the iterations.

3. Denoising using the improved wavelet threshold method

After denoising the grouped 3-D tensors using nonlocal low-rank tensor decomposition, we obtain the denoised RCI. However, there is still a certain amount of noise left, so it is necessary to further remove the residual noise to obtain better denoising results. In this step, we process the residual noise of the RCI using the improved wavelet threshold algorithm, which is processed by denoising each eigen-image in the RCI in turn.

The wavelet threshold algorithms have been proven to achieve good results in denoising 2-D images. Since Donoho et al. proposed the hard threshold [53] and soft threshold functions [54], many scholars have improved the threshold function by solving the problems of discontinuous image signals and the over-smoothing of images that exist in these two threshold functions, respectively. The new threshold function proposed by us not only solves the above problems but also has strong flexibility and adaptability because the threshold can change with the value of the high-frequency component of each layer in the wavelet decomposition.

The hard threshold and soft threshold functions proposed by Donoho are shown in Formulas (10) and (11), respectively.

(10) $${\tilde{W}}_{j,k}=\{\begin{array}{c} W_{j,k\;\;\;\;\;\;\;\;\;\;\;\;\;\;\;\;\;\;\;\;\;\;\;\;\;\;\;\;\;\;\;\;\;\;}\left|W_{j,k}\right|\geq T\\ 0\;\;\;\;\;\;\;\;\;\;\;\;\;\;\;\;\;\;\;\;\;\;\;\;\;\;\;\;\left|W_{j,k}\right|<T\; \end{array},$$

(11) $${\tilde{W}}_{j,k}=\{\begin{array}{c} sign(W_{j.k}){\left(\left|W_{j,k}\right|-T\right)}_{\;\;\;\;\;\;\;\;\;\;\;\;}\left|W_{j,k}\right|\geq T\\ 0\;\;\;\;\;\;\;\;\;\;\;\;\;\;\;\;\;\;\;\;\;\;\;\;\;\;\;\;\left|W_{j,k}\right|<T\; \end{array},$$

where $W_{j,k}$ is the *k*-th wavelet coefficient at the *j*-th scale of the noisy image after wavelet decomposition; ${\tilde{W}}_{j,k}$ is the wavelet coefficients obtained after threshold processing; T is the threshold; and *sign* is the sign function.

The following is the improved wavelet threshold function:

(12) $${\tilde{W}}_{j,k}=\{\begin{array}{c} sign(W_{j.k})\left(\left|W_{j,k}\right|-\frac{2\left(1-\omega \right)T}{1+e^{\alpha {\left(\left|W_{j,k}\right|-T\right)}^{z}}}\right)\;{\;}_{\;\;\;\;\;\;\;\;\;\;\;\;}\left|W_{j,k}\right|\geq T\\ sign\left(W_{j,k}\right)\omega \frac{{\left|W_{j,k}\right|}^{2}}{T}\;\;\;\;\;\;\;\;\;\;\;\;\;\;\;\;\;\;\;\;\;\;\;\;\;\;\left|W_{j,k}\right|<T\; \end{array},$$

where ω , α , and z are adjustable parameters. When $\left|W_{j,k}\right|<T$ , the threshold function is expressed as a quadratic function, which avoids the signal oscillation caused by the threshold function being set to 0 directly and solves the problem in which the wavelet coefficients of the traditional threshold function have a constant deviation with the estimated wavelet coefficients.

The selection of the threshold is also very important; too large a threshold will filter out some useful information in the image and too small a threshold will leave much noise [51].

The threshold proposed by Donoho is shown as follows [53]:

(13) $$T=\sigma \sqrt{2\ln\left(M\times N\right)},$$

where σ denotes the noise standard deviation and M×N denotes the size of the image.

The threshold in the improved algorithm is set as:

(14) $$T=\frac{\sigma \sqrt{2\ln\left(M\times N\right)}}{m\times j-1},$$

where m is an adjustable parameter, j(1≤j≤n) is the corresponding wavelet decomposition scale, σ is the standard deviation of the noise and is defined as: $\sigma =\frac{median\left(\left|W_{1,k}\right|\right)}{0.6745}$ , median(x) represents the calculation of the median value, $median\left(\left|W_{1,k}\right|\right)$ is the median of the absolute value of the wavelet decomposition coefficient of the first layer, and 0.6745 is the adjustment coefficient of the standard deviation of Gaussian noise [51].

The improved threshold formula can obtain the adaptive threshold according to the current value of the wavelet decomposition scale, and the threshold satisfies the requirement of gradually decreasing with the increase in decomposition scale.

### 3.3. Updating Orthogonal Basis and Representation Coefficients

Using the above operation, the denoising of RCI is completed, and the estimated clean 3-D tensors $C_{ind}$ is obtained. At this time, the denoising model becomes:

(15) $$\underset{\;\;\;\;\;\;\;\;\;\;\;\;\;\;\;\;\;\;\;\;E_{i},Z_{i},S_{i}}{\left\{{\hat{E}}_{i},{\hat{Z}}_{i},{\hat{S}}_{i}\right\}=arg\;min}\frac{1}{2}{\left||E_{i}^{T}Y_{i}-Z_{i}-E_{i}^{T}S_{i}|\right|}_{F}^{2}+\lambda_{1}\sum_{ind}\left(\frac{1}{\sigma_{ind}^{2}}{\left||G_{ind}Z_{i}-C_{ind}|\right|}_{F}^{2}\right)+\lambda_{2}||S_{i}{\left|\right|}_{1},\;\;\;\;\;\;\;\;s.t.\;\;E^{T}E=I_{k}$$

Because the denoised image $X_{i}=E_{i}Z_{i}$ obtained in this round of iteration will be used as the input for the new noisy HSI in the next round, to obtain a better denoising performance, the orthogonal basis Ε and the representation coefficients Z need to be updated. The ADMM algorithm [55] is used to update these variables with the basic idea of alternating optimization.

1. Updating $S_{i}$ by fixing $E_{i}$ and $Z_{i}$

The formula for optimizing the variable $S_{i}$ from the model (15) is as follows:

(16) $$\;{\hat{S}}_{i}=\underset{S_{i}}{\arg}\min\frac{1}{2}{\left||E_{i}^{T}Y_{i}-Z_{i}-E_{i}^{T}S_{i}|\right|}_{F}^{2}+\lambda_{2}||S_{i}{\left|\right|}_{1}=S_{\lambda_{2}}\left(Y_{i}-E_{i}Z_{i}\right)=sgn\left(Y_{i}-E_{i}Z_{i}\right)max\left(\left|Y_{i}-E_{i}Z_{i}\right|-\lambda_{2\;},0\right)$$

Actually, $S_{\lambda_{2}}\left(Y_{i}-E_{i}Z_{i}\right)$ is the soft threshold operator proposed by Donoho [54].

2. Updating $Z_{i}$ by fixing $E_{i}$ and $S_{i}$

The formula for optimizing the variable $Z_{i}$ from the model (15) is as follows:

(17) $$\begin{array}{c} {\hat{Z}}_{i}=\underset{Z_{i}}{\arg}\min\frac{1}{2}{\left||E_{i}^{T}Y_{i}-Z_{i}-E_{i}^{T}S_{i}|\right|}_{F}^{2}+\lambda_{1}\underset{ind}{\sum }\left(\frac{1}{\sigma_{ind}^{2}}{\left||G_{ind}Z_{i}-C_{ind}|\right|}_{F}^{2}\right)\\ =\left(\lambda_{1}\sum_{ind}V_{ind}+{E_{i}}^{T}\left(Y_{i}-S_{i}\right)\right)/(\lambda_{1}\sum_{ind}W_{ind}+I_{k}), \end{array}$$

3. Updating $E_{i}$ by fixing $Z_{i}$ and $S_{i}$

The formula for optimizing the variable $E_{i}$ from the model (15) is as follows:

(18) $$\underset{\;\;\;\;E_{i},\;{E_{i}}^{T}E_{i}=I_{k}}{{\hat{E}}_{i}=\arg}\min\frac{1}{2}{\left||E_{i}^{T}Y_{i}-Z_{i}-E_{i}^{T}S_{i}|\right|}_{F}^{2}=U\left(\alpha \right)V{\left(\alpha \right)}^{T},$$

where matrix $\alpha =\left(Y_{i}-S_{i}\right)\times {Z_{i}}^{T}$ , U(α) and V(α) represent the left singular vector and the right singular vector obtained from the SVD decomposition of matrix α , respectively.

### 3.4. Iterative Regularization

Iteration is often used in various algorithms to enhance their algorithmic performance [56,57]. Through the previous three steps, the algorithm realizes one round of denoising for the noisy HSI. To make the denoised HSI closer to the clean HSI, the algorithm continues to iteratively optimize the denoised HSI: $X_{i}$ . After repeated experiments, we found that updating the next round of input noisy HSI $Y_{i+1}$ as follows has a better denoising effect than using $X_{i}$ as the input HSI directly:

(19) $$Y_{i+1}=\mu X_{i}+\left(1-\mu \right)Y,$$

where μ∈(0,1) is used to balance the ratio of denoised HSI $X_{i}$ and original noisy HSI Y . Adding a certain proportion of original noisy HSI can motivate the algorithm to play a better denoising performance.

In addition, the subspace dimension k is updated using the iterative regularization, as well. We know that the more severe the noise corruption, the smaller the subspace dimension of the HSI, which means that the number of column vectors of the orthogonal basis used to compose the subspace from the decomposition of the noisy HSI is smaller [44]. As the noise variance of the HSI decreases gradually with the iterative denoising, the subspace dimension k increases gradually. So, when the subspace dimension of the original noisy HSI Y is computed using the Hysime algorithm [50], we make an update of the subspace dimension k using the following formula:

(20) $$k_{i+1}=k_{i}+\rho ,$$

where ρ≥1 is an integer. Experiments have demonstrated that the denoised HSI obtained by updating  k via Formula (20) has a better denoising effect compared to the subspace dimension computed using the Hysime algorithm [50] in the iterations. The reason being that the Hysime algorithm only considers Gaussian noise, whereas realistic HSIs are often accompanied by a variety of noises simultaneously [45].

The proposed algorithm in this paper is shown in Algorithm 1:
**Algorithm 1: HSI Denoising with the SNLTAI algorithm**Input: The noisy HSI 
Y
, the patch size 
s
, the number of similar 3-D image patches 
n
, the regularization parameters 
$\lambda_{1}$
 and 
$\lambda_{2}$
, the number of iterations *iter*, the parameters 
μ
 and 
ρ
, the wavelet basis, the decomposition scale 
j
, the adjustable parameters 
ω

, α

, z

, m
.Output: The final denoised HSI 
X
1. 
$X_{1}=Y_{1}=Y$
, estimate 
$k_{1}$
 by using the Hysime algorithm;2. for *i* = 1,2,3,…, *iter* do3.   Step of spectral low-rank:   Obtain orthogonal basis 
$E_{i}$
 and representation coefficient 
$Z_{i}$
 via SVD on 
$Y_{i}$
;4.   Step of spatial nonlocal similarity:   (1) Group similar 3-D image patches to construct 3-D tensor 
$G_{ind}Z_{i}$
;   (2) Denoise RCI via low-rank tensor decomposition;    (3) Denoise RCI via the improved wavelet algorithm;5.   Update Orthogonal basis 
$E_{i}$
 and representation coefficient 
$Z_{i}$
 with ADMM;6.   Iterative regularization:   (1) Update
$\;Y_{i+1}=\mu X_{i}+\left(1-\mu \right)Y$
;   (2) Update 
$k_{i+1}=k_{i}+\rho$
;7. end for8. Return the final denoised HSI 
X
;

## 4. Experiments and Analysis

In order to validate the effectiveness of the algorithm proposed in this paper, we conducted comparative experiments on the simulated and the real datasets of his. Nine image denoising algorithms were added to our comparative experiments, including BM4D [58], LRTV [34], LRMR [33], FastHyDe [41], GLF [42], NGmeet [44], LRTDTV [35], FastHyMix [43], and SNLRSF [45], and the parameter settings involved in these algorithms are consistent with their original papers. All the algorithms were run on MATLAB 2019a using a Lenovo computer with an Intel Core i5-6200U CPU and 8 GB of RAM. In addition, all the HSI datasets were normalized for each band before the experiment.

### 4.1. Simulated HSI Experiments

**Figure 2:**
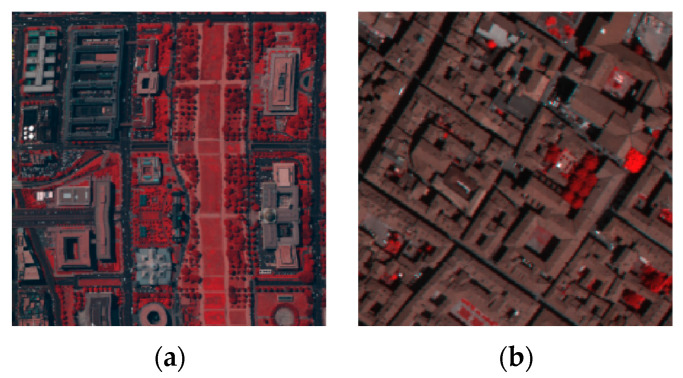
False color images of (**a**) WDC dataset (R:60 G:27 B:17); (**b**) Pavia C dataset (R:68 G:24 B:19).

Two simulated datasets of HSI were used in this experiment, including the Washington DC (WDC) dataset and the Pavia Center (Pavia C) dataset. The WDC dataset contains 191 bands, and the image size of each band is 1208 × 307 pixels. We selected 256 × 256 pixels of the bands as the experimental objects, so the sub-image of the experiment was 256 × 256 × 191. There are 102 bands in the Pavia C dataset, each band with a size of 1096 × 715 pixels. Only the last 80 bands were selected to simulate a clean dataset because the first 22 bands contain a large amount of noise. We intercepted 201 × 201 pixels as the experiment object, so the sub-image of the experiment was 201 × 201 × 80. The false color images of these two datasets are shown in Figure 2.

To simulate different noise environments, we added Gaussian noise, impulse noise, and stripe noise with different intensities to these two HSI datasets. The specific addition method was as follows:

1. For the WDC dataset

Case 1: Add zero-mean Gaussian noise with a standard deviation of 0.1 for each band.

Case 2: Randomly add zero-mean Gaussian noise with a standard deviation of [0.1,0.2] for each band.

Case 3: Based on Case 2, 45 bands are randomly selected to add impulse noise (salt and pepper noise) with a density of 20%.

Case 4: Continue to add stripe noise with intensity 0.3 to each band based on Case 3.

2. For the Pavia C dataset

Case 1: Add zero-mean Gaussian noise with a standard deviation of 0.2 for each band.

Case 2: Randomly add zero-mean Gaussian noise with a standard deviation of [0.1,0.2] for each band.

Case 3: Based on Case 2, impulse noise (salt and pepper noise) is added to each band with a density of 40%.

Case 4: Continue to add stripe noise with an intensity of 0.3 to each band based on Case 3.

In addition, five commonly used metrics were used to comprehensively evaluate the denoised results of various algorithms accurately and objectively. They are the mean peak signal-to-noise ratio (MPSNR), the mean structural similarity (MSSIM) [59], the error relative global adimensionnelle de synthese (ERGAS) [60], the mean spectral angle (MSA), and the spectral angle mapper (SAM) [61]. The larger the values of MPSNR and MSSIM, the better the image quality. Conversely, the smaller the values of the ERGAS, MSA, and SAM, the better the image quality. In addition, the running time of each algorithm is also included in the overall evaluation of the algorithm’s performance.

The formulas for these five evaluation metrics are given below.

(21) $$MPSNR=\frac{1}{l}\overset{l}{\underset{i=1}{\sum }}psnr_{i}$$

(22) $$MSSIM=\frac{1}{l}\overset{l}{\underset{i=1}{\sum }}ssim_{i}$$

(23) $$ERGAS=\sqrt{\frac{1}{l}\sum_{i=1}^{l}\frac{mse\left(ref_{i},den_{i}\right)}{Mean_{2}\left(ref_{i}\right)}}$$

(24) $$MSA=\frac{1}{mn}\overset{m\;n}{\underset{i=1}{\sum }}\frac{180}{\pi }\times arccos\frac{{\left(\chi^{i}\right)}^{T}\cdot \left({\hat{\chi }}^{i}\right)}{\left||\chi^{i}||\cdot ||{\hat{\chi }}^{i}|\right|}$$

(25) $$SAM=arccos\left(\frac{\langle u_{R},u_{F}\rangle }{||u_{R}{\left|\right|}_{2}\cdot ||u_{F}{\left|\right|}_{2}}\right)$$

where $psnr_{i}$ and $ssim_{i}$ represent the PSNR and SSIM values for the i th band, $ref_{i}$ and $den_{i}$ denote the band (spectral signatures) of the original noisy HSI and the denoised HSI, respectively. In Formula (23), $Mean_{2}\left(ref_{i}\right)$ denotes the average pixel value for the $ref_{i}$ . In Formula (25), $\;u_{R}$ and $u_{F}$ denote the spectral vectors of the original noisy HSI and the denoised HSI, respectively.

Table 1 and Table 2 show the metric values obtained from the WDC dataset and Pavia C dataset, respectively, using different noise addition methods in different denoising algorithms. The best metric values are shown in bold, and the second-best metric values are shown in italics. The data in the tables demonstrate that compared with the state-of-the-art HSI denoising algorithms, the SNLTAI algorithm has great advantages in each evaluation metric, which proves that the algorithm has excellent denoising performance for different noise types and noise intensity. In terms of running time, the SNLTAI algorithm takes less time and has a lower computational cost, except for the FastHyDe and the FastHyMix algorithms. As mentioned above, short running time and fast speed are the greatest advantages of FastHyMix algorithm.

**Figure 3:**
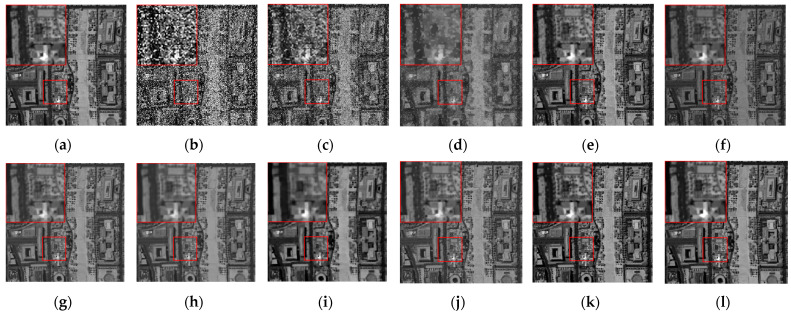
Denoised images of different algorithms on the 95th band of the WDC dataset (CASE3): (**a**) Original; (**b**) Noisy; (**c**) BM4D; (**d**) LRTV; (**e**) LRMR; (**f**) FastHyDe; (**g**) GLF; (**h**) NGmeet; (**i**) LRTDTV; (**j**) FastHyMix; (**k**) SNLRSF; and (**l**) SNLTAI.

**Figure 4:**
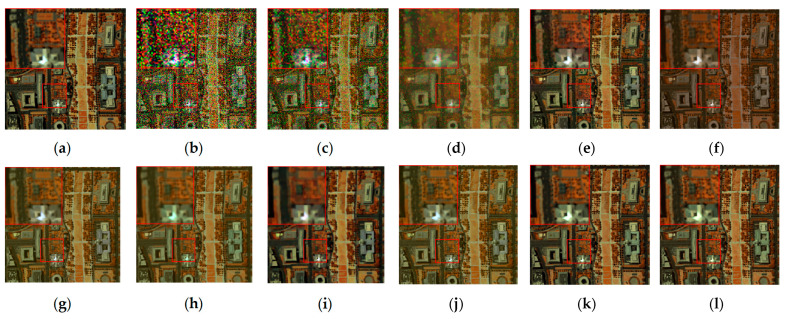
Denoised false color images (R:95, G:114, B:153) of different algorithms of the WDC dataset (CASE3): (**a**) Original; (**b**) Noisy; (**c**) BM4D; (**d**) LRTV; (**e**) LRMR; (**f**) FastHyDe; (**g**) GLF; (**h**) NGmeet; (**i**) LRTDTV; (**j**) FastHyMix; (**k**) SNLRSF; and (**l**) SNLTAI.

**Figure 5:**
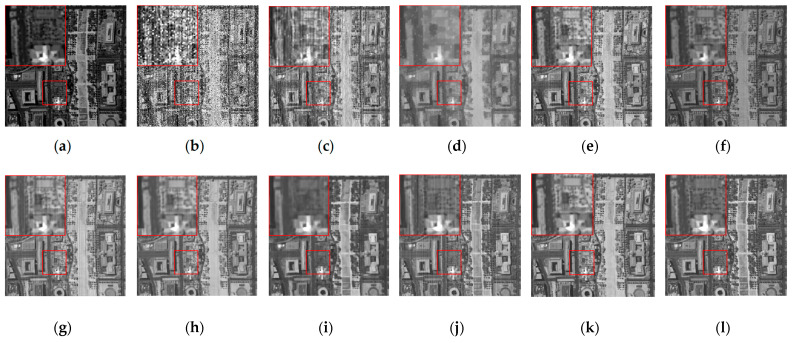
Denoised images of different algorithms on the 114th band of the WDC dataset (CASE4): (**a**) Original; (**b**) Noisy; (**c**) BM4D; (**d**) LRTV; (**e**) LRMR; (**f**) FastHyDe; (**g**) GLF; (**h**) NGmeet; (**i**) LRTDTV; (**j**) FastHyMix; (**k**) SNLRSF; and (**l**) SNLTAI.

**Figure 6:**
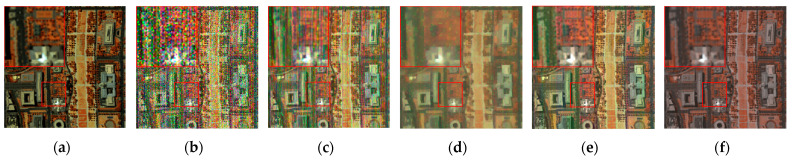

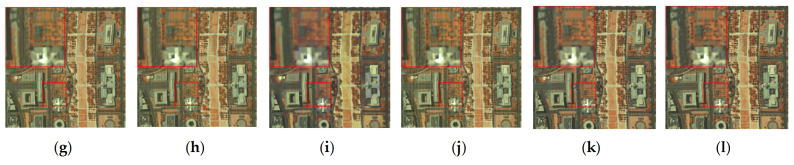
Denoised false color images (R:95, G:114, B:153) of different algorithms of the WDC dataset (CASE4): (**a**) Original; (**b**) Noisy; (**c**) BM4D; (**d**) LRTV; (**e**) LRMR; (**f**) FastHyDe; (**g**) GLF; (**h**) NGmeet; (**i**) LRTDTV; (**j**) FastHyMix; (**k**) SNLRSF; and (**l**) SNLTAI.

**Figure 7:**
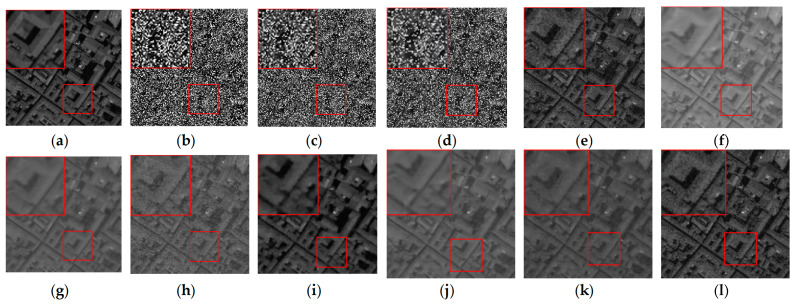
Denoised images of different algorithms on 34th band of the Pavia C dataset (CASE3): (**a**) Original; (**b**) Noisy; (**c**) BM4D; (**d**) IV; (**e**) LRMR; (**f**) FastHyDe; (**g**) GlF; (**h**) NGmeet; (**i**) LRTDTV; (**j**) FastHyMix; (**k**) SNLRSF; and (**l**) SNLTAI.

**Figure 8:**
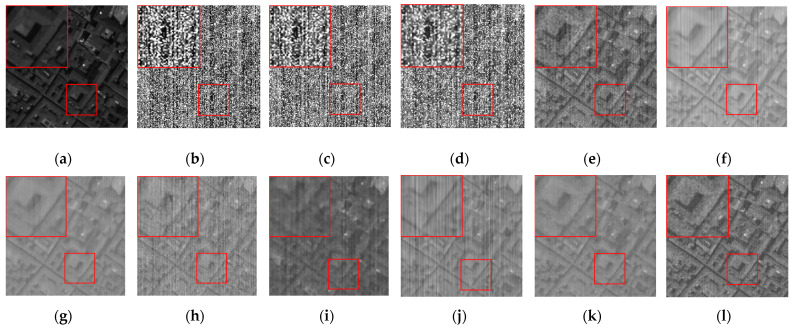
Denoised images of different algorithms on the 6th band of the Pavia C dataset (CASE4): (**a**) Original; (**b**) Noisy; (**c**) BM4D; (**d**) LRTV; (**e**) LRMR; (**f**) FastHyDe; (**g**) GLF; (**h**) NGmeet; (**i**) LRTDTV; (**j**) FastHyMix; (**k**) SNLRSF; and (**l**) SNLTAI.

The denoising results of different algorithms for the WDC and Pavia C datasets in CASE3 and CASE4 are shown in Figure 3, Figure 4, Figure 5, Figure 6, Figure 7 and Figure 8. We use a small red box to mark the detail-rich parts of the images and then use a large red box to enlarge the part in the upper left corner of the image to show and compare the noise removal visual performance of each algorithm more clearly.

Figure 3 shows the denoised images of the 95th band of the WDC dataset with Gaussian noise and impulse noise added to CASE3. Figure 4 shows the denoised false color images (R:95, G:114, B:153) of different algorithms of the WDC dataset on CASE3. Figure 5 shows the denoised images of the 114th band of the WDC dataset with Gaussian noise, impulse noise, and stripe noise added to CASE4, and Figure 6 shows the denoised false color images (R:95, G:114, B:153) of different algorithms of the WDC dataset on CASE4. The noisy images of CASE3 and CASE4 demonstrate that the original images are severely polluted. BM4D and LRTV algorithms cannot completely remove all the noise, and there are still many noise points and patches left. The GLF and FastHyMix algorithms are not thorough enough to remove the stripe noise. Other algorithms can remove the noise to a certain extent, but in terms of the recovery of the images’ textures and details, the SNLTAI algorithm performs better. The denoised false color images also reflect that SNLTAI performs better compared to other algorithms, both in terms of color reproduction and the presentation of image details and textures.

Figure 7 shows the denoising effect of each algorithm on CASE3 in the 34th band of the Pavia C dataset, and Figure 8 shows the denoising effect of each algorithm on CASE4 in the 6th band of the Pavia C dataset. As demonstrated in the images, the 34th and the 6th bands are severely polluted by various kinds of noise, and the noisy images have completely failed to reflect any shape or details of the objects in the original images. The BM4D and LRTV algorithms are unable to fix the serious noise damage, and there are still obvious noise points and stripes in the denoised image of the LRMR algorithm. For the FastHyDe, GLF, NGmeet, LRTDTV, and FastHyMix algorithms, the removal of severe stripe noise needs to be enhanced, and the SNLRSF algorithm loses too much of the image details. Comparatively, the SNLTAI algorithm performs much better in terms of removing the noise and keeping the details.

Therefore, the SNLTAI algorithm has a strong ability to remove all types of noise with different intensities, which also reflects the unique advantages of subspace and spectral low-rank decomposition in image denoising.

**Figure 9:**
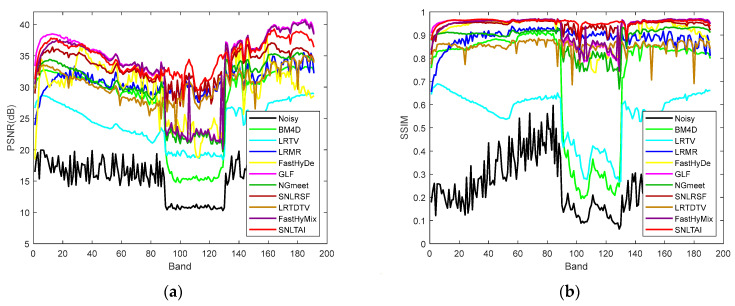

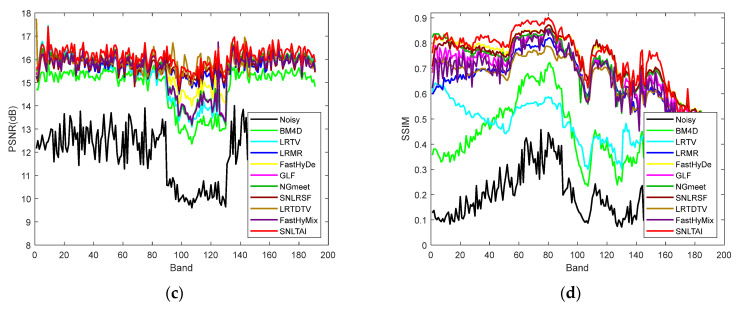
PSNR/SSIM of each denoised band in the WDC dataset with different algorithms: (**a**) PSNR_WDC_CASE3; (**b**) SSIM_ WDC_CASE3; (**c**) PSNR_ WDC_CASE4; and (**d**) SSIM_ WDC_CASE4.

**Figure 10:**
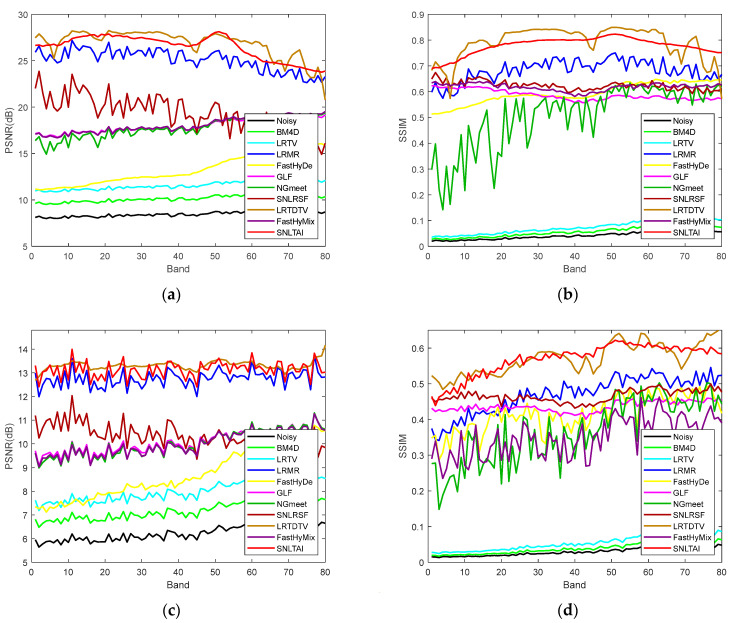
PSNR/SSIM of each denoised band in the Pavia C dataset with different algorithms: (**a**) PSNR_ Pavia C _CASE3; (**b**) SSIM_ Pavia C _CASE3; (**c**) PSNR_ Pavia C _CASE4; and (**d**) SSIM_ Pavia C _CASE4.

Figure 9 and Figure 10 show the comparison of PSNR and SSIM in each band of these two denoised HSI datasets using various algorithms. The comparison of the curves in the figures proves the denoising advantages of the SNLTAI algorithm again.

### 4.2. Real HSI Experiments

To further validate the denoising performance of the SNLTAI algorithm, we also conducted experiments on two real HSI datasets. Because the noise level needs to be estimated on the real datasets before denoising, we whitened the noise [42] or estimated the noise level by utilizing the algorithm from the reference [54] before running each algorithm.

#### 4.2.1. AVIRIS Indian Pines Dataset

**Figure 11:**
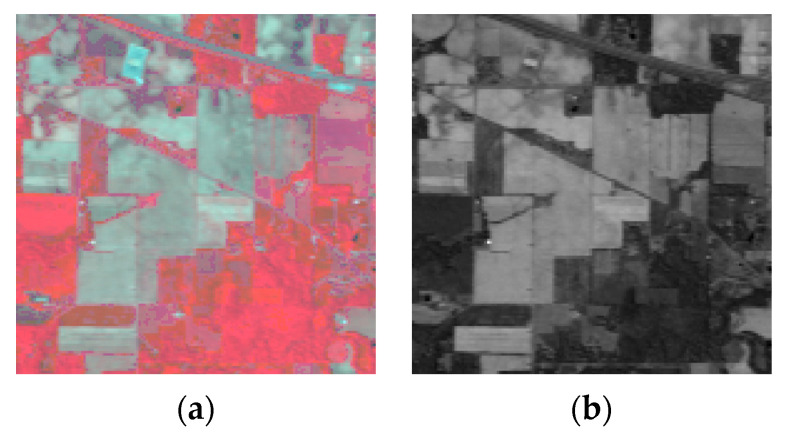
(**a**) False color images of the Indian Pines dataset (R:50, G:27, B:19); (**b**) The 145th band image of the Indian Pines dataset.

The first real dataset is the AVIRIS Indian Pines dataset, which was taken with the AVIRIS sensor in 1992 in Indiana, U.S.A. The size of the dataset is 145 × 145 × 220. Some bands of this dataset are contaminated with a mixture of Gaussian noise, impulse noise, stripe noise, and water absorption, etc. Figure 11 shows a false color image of this dataset (R:50, G:27, B:19) and the 145th band image.

**Figure 12:**
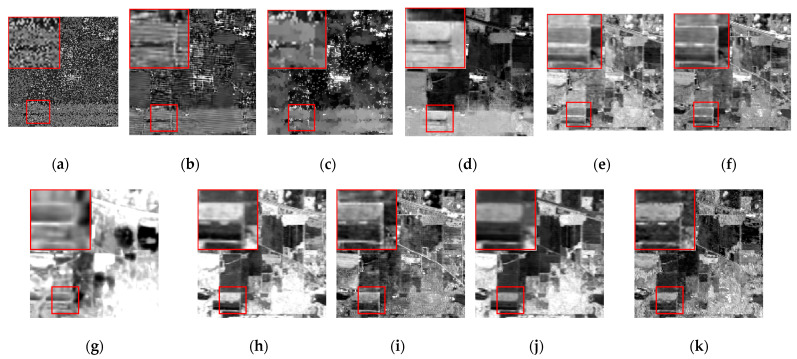
Denoised images of different algorithms on the 1st band of the Indian Pines dataset: (**a**) Original; (**b**) BM4D; (**c**) LRTV; (**d**) LRMR; (**e**) FastHyDe; (**f**) GLF; (**g**) NGmeet; (**h**) LRTDTV; (**i**) FastHyMix; (**j**) SNLRSF; and (**k**) SNLTAI.

**Figure 13:**
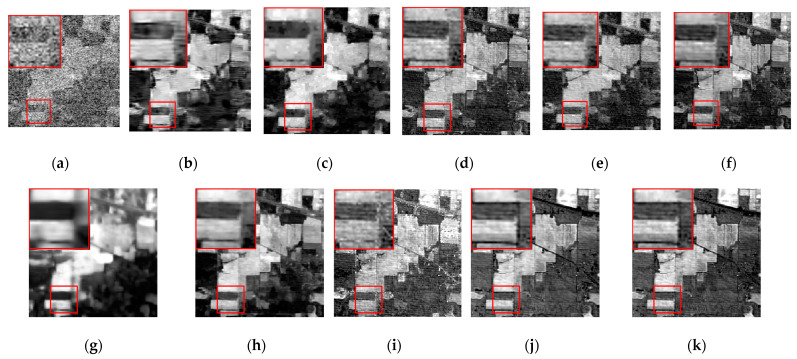
Denoised images of different algorithms on the 109th band of the Indian Pines dataset: (**a**) Original; (**b**) BM4D; (**c**) LRTV; (**d**) LRMR; (**e**) FastHyDe; (**f**) GLF; (**g**) NGmeet; (**h**) LRTDTV; (**i**) FastHyMix; (**j**) SNLRSF; and (**k**) SNLTAI.

We chose the 1st and 109th bands as the experimental images for the single-band denoising comparison of each algorithm, as shown in Figure 12 and Figure 13.

The band 1 image is heavily corrupted by various types of noise, and the image can reflect no useful information. It is not difficult to determine that the BM4D and LRTV algorithms can barely conduct effective noise removal to recover image details, and there is even the imprint of stripe noise in the BM4D algorithm. The LRMR algorithm also performs poorly against heavy pollution, with poor recovery of the red box in the image, which is lacking in texture and details. The NGmeet algorithm has issues with over-smoothing and serious detail loss. The contrast range of gray values in the whole image is too large, and the details are lost in both the bright and the dark parts of the image. The FastHyMix algorithm also retains pronounced stripe noise. Although the rest of the algorithms can recover the textures and details of the image to some extent, the SNLTAI algorithm is comparatively better at detail recovery and visual intuition.

The 109th band image is also severely corrupted by various types of noise, and only the general outline of the building can be seen in the image, and the details and textures are completely lost. All the algorithms can recover this band image to a certain extent. However, the BM4D, LRTV, and NGmeet algorithms result in unclear details and over-smoothing of the image, and the enlarged red boxes of the images processed by the LRMR, FastHyDe, GLF, and LRTDTV algorithms are somewhat blurry, with unclear boundaries. The FastHyMix algorithm also retains a significant residual noise distribution. Comparatively speaking, the images recovered using the SNLRSF and SNLTAI algorithms are closer to each other, and both of them recovered the detail and boundary information from the original image. This again shows that the SNLTAI algorithm can achieve excellent denoising performance because it utilizes both subspace representation and nonlocal low-rank tensor decomposition.

**Figure 14:**
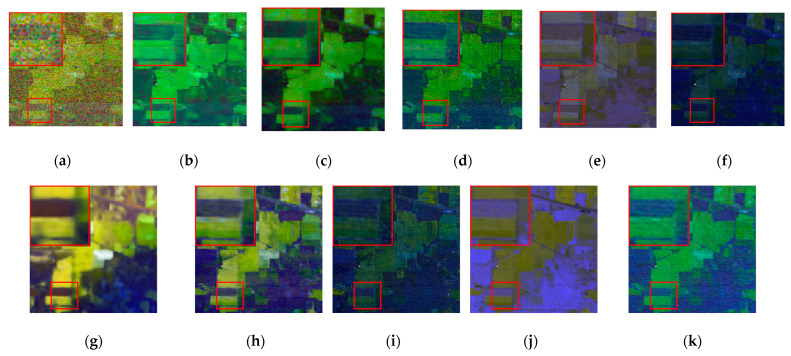
Denoised false color images (R:219, G:109, B:1) of different algorithms of the Indian Pines dataset: (**a**) Original; (**b**) BM4D; (**c**) LRTV; (**d**) LRMR; (**e**) FastHyDe; (**f**) GLF; (**g**) NGmeet; (**h**) LRTDTV; (**i**) FastHyMix; (**j**) SNLRSF; and (**k**) SNLTAI.

In order to further compare the denoising performance of various algorithms, we also performed false color composition on the denoised images of the Indian Pines dataset (R:219, G:109, B:1). In Figure 14, it is shown that BM4D and FastHyMix still have obvious horizontal stripes, LRTV and NGmeet have blurred images with lost details, and NGmeet is even worse, whereas FastHyDe, GLF, and SNLRSF do not perform well in color restoration. Relatively speaking, LRMR, LRTDTV, and SNLTAI perform better in denoising, color restoration, and detail presentation.

**Figure 15:**
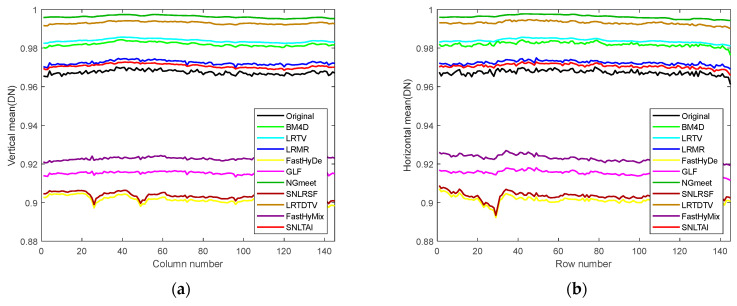
Mean profiles of band 150 of the Indian Pines dataset with different algorithms: (**a**) vertical mean profiles and (**b**) horizontal mean profiles.

In addition to analyzing and comparing the visual effects of the denoised images, we also provide the vertical and horizontal mean profiles of band 150 after processing it using different algorithms, as shown in Figure 15. The rapid fluctuation of the original curve indicates that the image contains a large amount of irregular noise [45], and the smooth curve after noise removal indicates that the algorithm has a strong noise removal ability. Meanwhile, the closer the average gray value of the curve after noise removal is to the original image, the more consistent the brightness of the denoised image is with that of the original image. It is not difficult to see from the figure that among all the algorithms, the SNLTAI algorithm performs the best in combining these two evaluation criteria, thus once again proving the advantages of the SNLTAI algorithm for HSI denoising.

#### 4.2.2. HYDICE Urban Dataset

**Figure 16:**
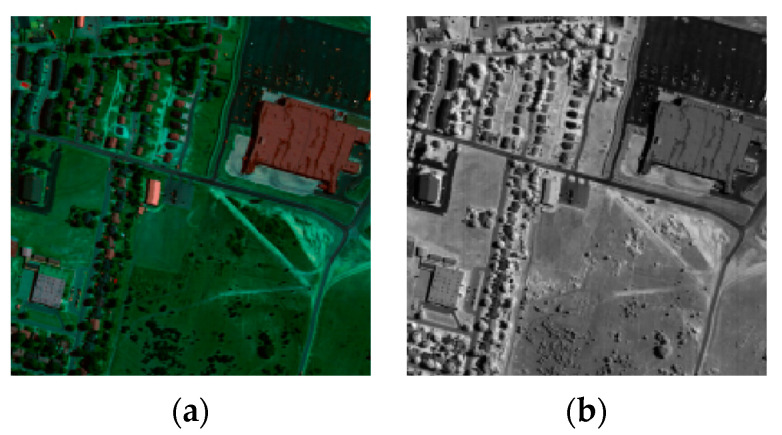
(**a**) False color images of the HYDICE Urban Dataset (R:16, G:118, B:153); (**b**) the 79th band image of the HYDICE Urban Dataset.

The original HYDICE Urban dataset was obtained from the HYDICE sensors with a size of 307 × 307 × 210. Because some bands are severely polluted by noise such as stripes, deadlines, and atmospheric and water absorption and cannot provide any valuable information, we finally chose a sub-image with a size of 200 × 200 × 162 as the final experimental object. Figure 16 shows a false color image (R:16, G:118, B:153) and the 79th band image of the Urban dataset.

**Figure 17:**
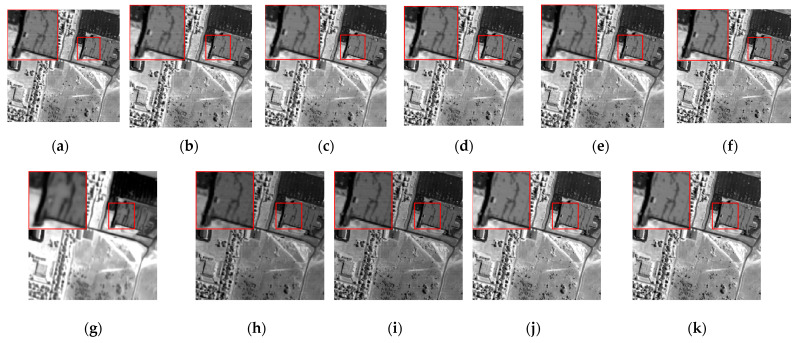
Denoised images using different algorithms on the 83rd band of the Urban dataset: (**a**) Original; (**b**) BM4D; (**c**) LRTV; (**d**) LRMR; (**e**) FastHyDe; (**f**) GLF; (**g**) NGmeet; (**h**) LRTDTV; (**i**) FastHyMix; (**j**) SNLRSF; and (**k**) SNLTAI.

Figure 17 shows the denoised images of the 83rd band processed using various algorithms. The original image contains a little Gaussian noise and a lot of horizontal stripe noise. The BM4D, LRTV, LRMR, FastHyDe, GLF, and FastHyMix algorithms have a weak ability to remove stripe noise, as the stripes can still be clearly seen in the image after noise removal. Although the NGmeet algorithm can effectively remove the stripes, the denoised image is too smooth, resulting in blurring and the loss of a large amount of detail. Although the LRTDTV algorithm removes the stripes, the overall image is darker, and the brightness and contrast of the original image are greatly changed. The SNLRSF and SNLTAI algorithms can completely remove the stripes, and the image details and textures are maintained quite well.

**Figure 18:**
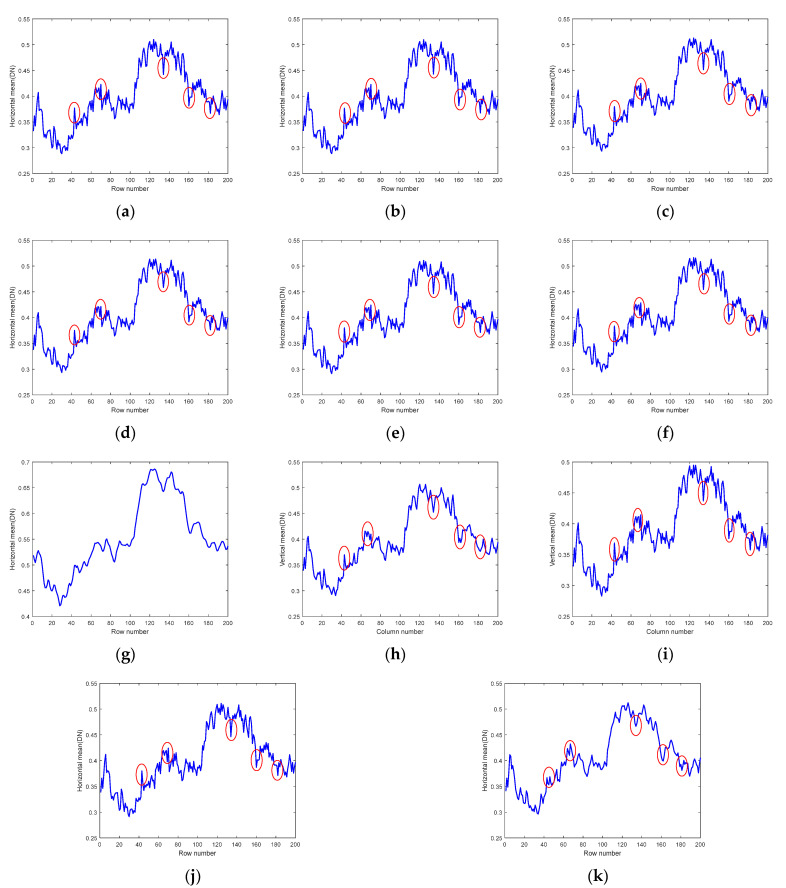
Horizontal mean profiles for the Urban dataset (Band 83): (**a**) Original; (**b**) BM4D; (**c**) LRTV; (**d**) LRMR; (**e**) FastHyDe; (**f**) GLF; (**g**) NGmeet; (**h**) LRTDTV; (**i**) FastHyMix; (**j**) SNLRSF; and (**k**) SNLTAI.

Moreover, we also provide the horizontal mean profiles of the 83rd band image before and after denoising using different algorithms, as shown in Figure 18. The rapid fluctuation of the curve in the original image reflects the existence of stripe noise [33], and we marked the obvious fluctuation of the curve with a red ellipse. The image curves demonstrate that the BM4D, LRTV, LRMR, FastHyDe, GLF, and FastHyMix algorithms have no significant changes in these fluctuations, indicating that their ability to remove stripe noise is extremely limited, which is also consistent with the effect reflected in the denoised images. Furthermore, although the curve of the NGmeet algorithm does not contain these fluctuations, the curve is too smooth, indicating that much detail is lost; even the average DN value is far beyond the normal range. It can be inferred that the overall gray value of the denoised image is much larger than that of the original image, and the visual reflection is that the overall image is too bright, which is also consistent with the information reflected in the denoised image. The LRTDTV algorithm can remove these fluctuations to some extent. However, the SNLRSF and SNLTAI algorithms almost completely eliminate these fluctuations, and the curves are overall smoother, indicating that the stripe noise in this band is effectively removed.

**Figure 19:**
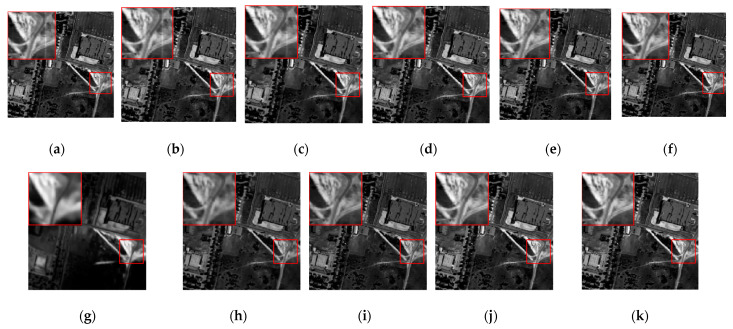
Denoised images of different algorithms on the 121st band of the Urban dataset: (**a**) Original; (**b**) BM4D; (**c**) LRTV; (**d**) LRMR; (**e**) FastHyDe; (**f**) GLF; (**g**) NGmeet; (**h**) LRTDTV; (**i**) FastHyMix; (**j**) SNLRSF; and (**k**) SNLTAI.

**Figure 20:**
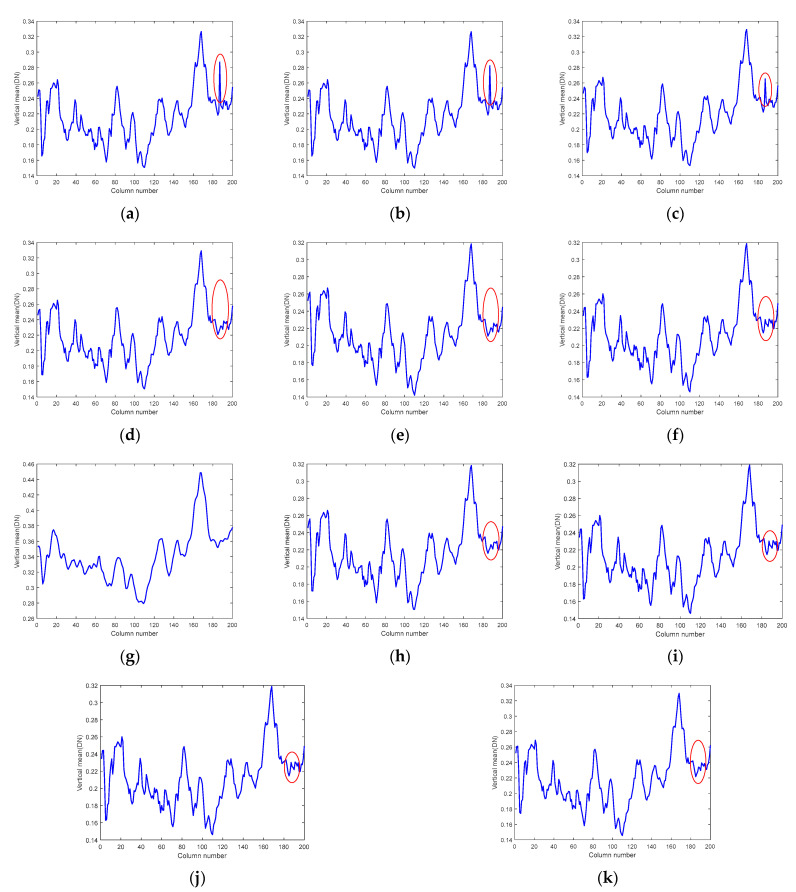
Vertical mean profiles for the Urban dataset (Band 121): (**a**) Original; (**b**) BM4D; (**c**) LRTV; (**d**) LRMR; (**e**) FastHyDe; (**f**) GLF; (**g**) NGmeet; (**h**) LRTDTV; (**i**) FastHyMix; (**j**) SNLRSF; and (**k**) SNLTAI.

Figure 19 and Figure 20 provide the image comparison and the vertical mean profiles of the 121st band before and after denoising. We can see that stripe noise still exists in band 121, and there is also a noticeable deadline in the red box. The denoised images and profiles reflect that the BM4D algorithm cannot successfully remove stripe noise and deadlines; the LRTV algorithm can suppress stripe noise effectively, but the deadlines still exist; and the LRMR, FastHyDe, GLF, and FastHyMix algorithms can filter the deadlines, but there is a certain degree of stripe noise left. The LRTDTV algorithm removes deadlines and stripes, but still reduces the contrast of the image, and the image is somewhat blurred, losing certain textures and details. The NGmeet algorithm still has the problem that the denoised image is too smooth, resulting in the loss of detail, and the overall gray value of the denoised image becomes larger. Both the SNLRSF and SNLTAI algorithms can completely suppress the stripe noise and deadlines. However, from the profiles, the curve of the SNLTAI algorithm is relatively smoother, which shows that the SNLTAI algorithm has a stronger denoising ability and better denoising performance.

### 4.3. Parameters Analysis

In this section, we analyze and discuss the parameters in the SNLTAI algorithm to find their most appropriate values. We extracted a CASE each from the WDC dataset and the Pavia C dataset for the corresponding analysis and discussion. MPSNR and MSSIM were used as evaluation metrics for parameter analysis.

1. Regularization parameters $\lambda_{1}$ and $\lambda_{2}$

**Figure 21:**
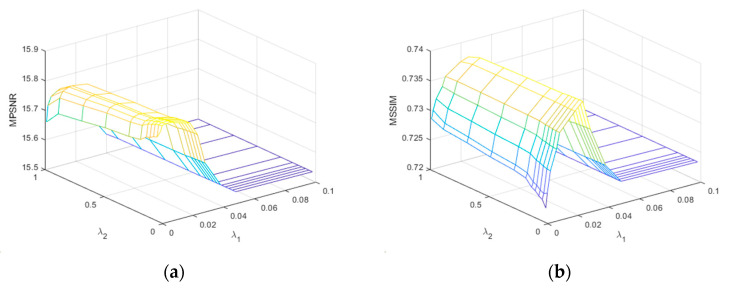
Parameter analysis for $\lambda_{1}$ and $\lambda_{2}$ in CASE4 of the WDC dataset: (**a**) MPSNR and (**b**) MSSIM.

**Figure 22:**
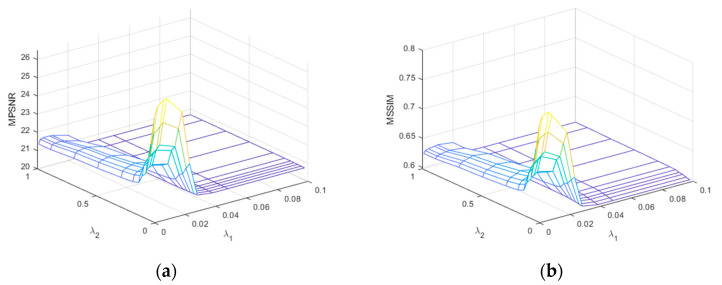
Parameter analysis for $\lambda_{1}$ and $\lambda_{2}$ in CASE3 of the Pavia C dataset: (**a**) MPSNR and (**b**) MSSIM.

CASE4 of the WDC dataset and CASE3 of the Pavia C dataset were extracted as the objects for parameter analysis. We selected $\lambda_{1}$ from the set of {0.001,0.002,0.004,0.008,0.01,0.02,0.025,0.03,0.04,0.05,0.1}, and $\lambda_{2}$ from the set of {0.03,0.06,0.09,0.12,0.15,0.2,0.3,0.5,0.7,0.9,1.0}. As demonstrated in Figure 21 and Figure 22, although the surface shapes are different, the MPSNR and MSSIM of both WDC and Pavia C reach their highest values around $\lambda_{1}\;$ = 0.02 and then gradually decrease until they become stable. Therefore, without loss of generality, $\lambda_{1}$ = 0.02 and $\lambda_{2}$ = 0.06 were selected as the parameter values of the simulated and the real datasets of the SNLTAI algorithm.

2. Analysis of the number of iterations and algorithm convergence

**Figure 23:**
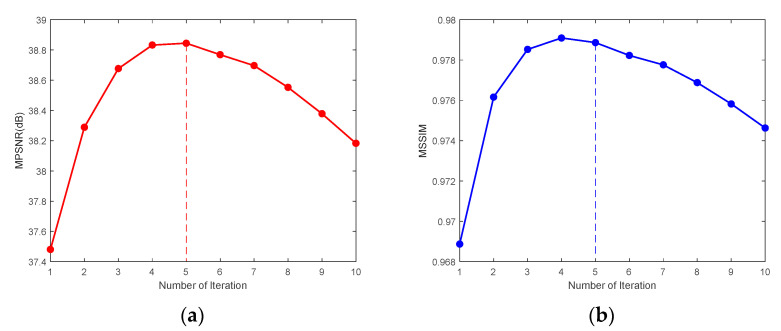
Analysis of the number of iterations for the WDC dataset CASE1: (**a**) MPSNR and (**b**) MSSIM.

**Figure 24:**
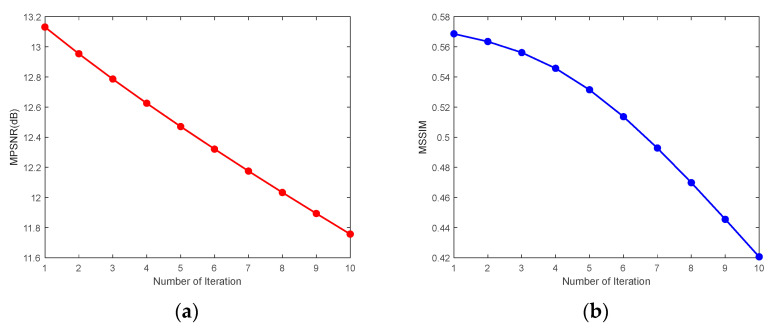
Analysis of the number of iterations for the Pavia C dataset CASE4: (**a**) MPSNR and (**b**) MSSIM.

We selected CASE1 of the WDC dataset and CASE4 of the Pavia C dataset for this study of iteration number *Iter*. Figure 23 demonstrates that for the WDC dataset CASE1, when the noise intensity is relatively small, the best results of denoising can be obtained when the number of iterations is 4 or 5, and in the algorithm, we take *Iter* = 5. Figure 24 reflects the iteration of the Pavia C dataset CASE4. It is clear that the best effect of denoising is achieved after the first iteration because the noise intensity of CASE4 is very high, and the textures and details of the original image are completely invisible. Repeated denoising processes will lead to the deviation between the denoised image and the original image increasing. Therefore, for CASE3 and CASE4 with extremely high noise intensity, the number of iterations was set to 1. In addition, Figure 23 and Figure 24 demonstrate that as the number of iterations gradually increases, the denoising effect decreases slowly until it converges to a certain value.

**Figure 25:**
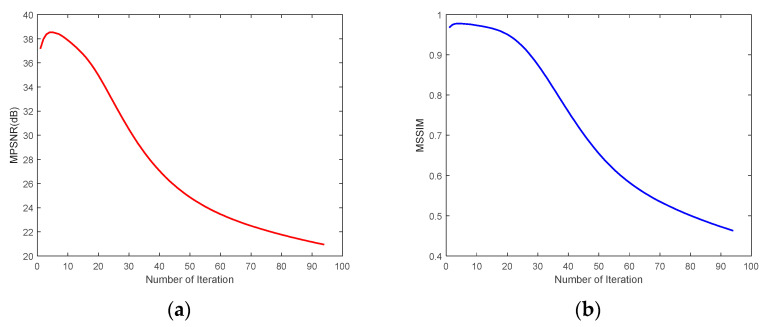
Convergence analysis for the WDC dataset CASE1: (**a**) MPSNR and (**b**) MSSIM.

**Figure 26:**
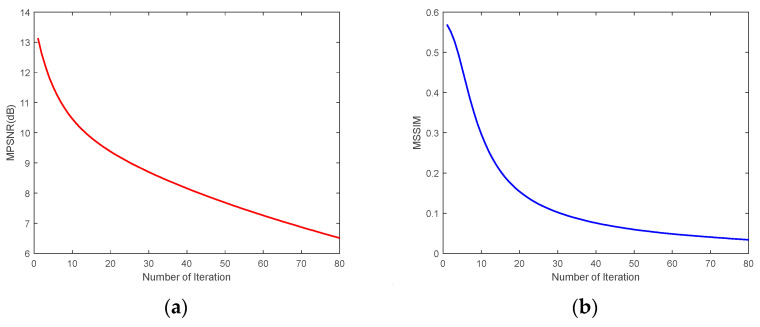
Convergence analysis for the Pavia C dataset CASE4: (**a**) MPSNR and (**b**) MSSIM.

To further illustrate the convergence of the SNLTAI algorithm, the trend plots of the gradual convergence of MPSNR and MSSIM as the number of iterations increases for the CASE1 of the WDC dataset and the CASE4 of the Pavia C dataset are given in Figure 25 and Figure 26, respectively.

3. The number of nonlocal similar 3-D image patches n

**Figure 27:**
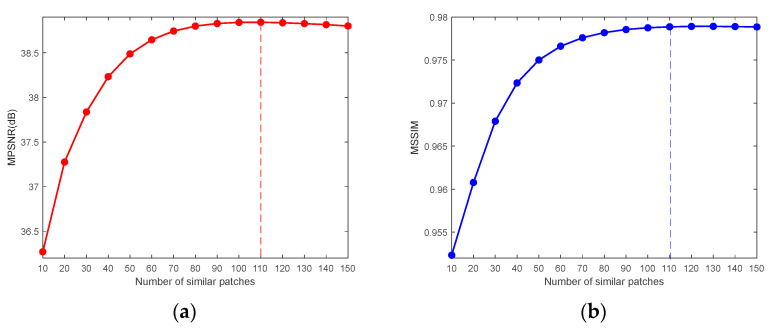
Analysis of the number of similar 3-D patches for the WDC dataset CASE1: (**a**) MPSNR and (**b**) MSSIM.

**Figure 28:**
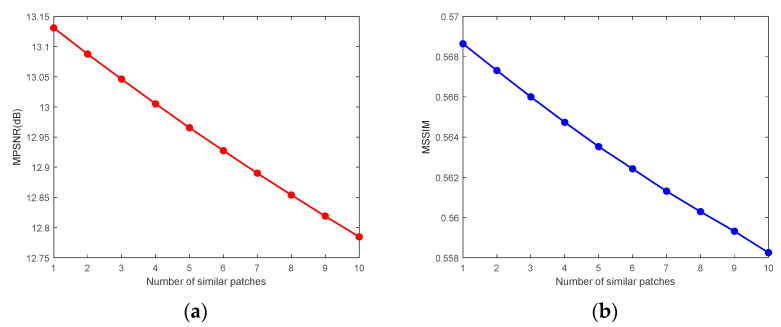
Analysis of the number of similar 3-D patches for the Pavia C dataset CASE4: (**a**) MPSNR and (**b**) MSSIM.

We selected the CASE1 of the WDC dataset and the CASE4 of the Pavia C dataset to study the number of similar 3-D patches. Because the CASE1 noise intensity of the WDC dataset is not high, a larger number of 3-D image patches is more likely to help the 3-D reference patch match and group similar image patches, thus improving the denoising performance. Figure 27 demonstrates that when the number of similar 3-D patches reaches 110, MPSNR and MSSIM reach the maximum value at almost the same time, and the values of these two metrics slowly decrease with the gradual increase in the number of similar 3-D patches. Therefore, for CASE1 and CASE2 of these two datasets, we take the value of n = 110. In contrast, Figure 28 reflects that in the case of extremely high noise intensity, the fewer the similar 3-D patches, the more favorable the denoising. Because the more similar the 3-D patches, the more they deviate from the textures and details of the reference patch, the best denoising effect can be achieved in this case by setting the number of similar 3-D image patches to n = 1.

4. For the wavelet threshold denoising function in the proposed algorithm, the wavelet basis is set as *sym15*, the decomposition scale is set to j = 1, and the adjustable parameters are set to ω = 0.3,  α = 21,  z = 3.09,  m = 2. After many experiments, the value of parameter μ in Formula (20) is set as μ = 0.95, and ρ in Formula (21) is set as ρ = 2 in both the simulated and real dataset experiments.

## 5. Conclusions

In this paper, a new HSI denoising algorithm based on subspace and nonlocal low-rank tensor decomposition is proposed to remove mixed noise, and the algorithm takes full advantage of the spectral low-rank and spatial nonlocal self-similarity of HSIs. Because the valid information in HSIs exists in a low-dimensional subspace, denoising HSI can be converted to denoising subspace, which greatly saves the time cost of the algorithm. Not only does the subspace representation allow for spectral low rank, but spatial nonlocal self-similarity can also be realized in subspace. The algorithm performs low-rank tensor decomposition and an improved wavelet threshold method for denoising the tensor composed of similar 3-D image patches. Then the obtained image continues to be updated using the ADMM algorithm, and finally, the updated denoised HSI is subjected to iterative regularization to obtain the best denoising results. After comparing this algorithm with other typical HSI denoising algorithms using simulated and real HSI datasets, the results show that this algorithm has the best relative denoising performance in terms of both objective evaluation metrics and subjective visual reflection. In future research, we will further improve the algorithm and consider dynamically adjusting the values of the regularization parameters $\lambda_{1},\lambda_{2}$ , and the number of nonlocal similar 3-D image patches n with the number of iterations to achieve better denoising performance.

## Figures and Tables

**Table 1:** Comparison of denoising metrics of different algorithms on the WDC dataset.

WDC Dataset												
Noise Case	Index	Noisy Data	BM4D	LRTV	LRMR	FastHyDe	GLF	NGmeet	LRTDTV	FastHyMix	SNLRSF	SNLTAI
CASE1	MPSNR	20.00	32.88	25.74	34.23	34.90	*38.72*	38.68	33.57	38.25	38.39	**38.84**
	MSSIM	0.4218	0.9128	0.6762	0.9384	0.9654	0.9781	*0.9787*	0.9274	0.9736	0.9774	**0.9789**
	ERGAS	388.54	85.79	198.45	72.85	76.25	43.77	*43.66*	78.49	46.02	45.84	**42.94**
	MSA	0.4614	0.0933	0.2594	0.0921	0.0909	0.0480	**0.0468**	0.0750	0.0517	0.0502	*0.0472*
	SAM	26.44	5.35	14.86	5.28	8.69	*2.83*	*2.83*	4.29	2.96	3.20	**2.70**
	Time (s)		673	624	200	*38*	1066	160	337	**13**	770	103
CASE2	MPSNR	16.58	30.48	25.25	31.49	32.28	*36.73*	35.02	32.71	36.30	35.86	**36.77**
	MSSIM	0.2796	0.8558	0.6053	0.8937	0.9336	*0.9665*	0.9529	0.9124	0.9605	0.9631	**0.9672**
	ERGAS	589.78	112.43	206.59	99.87	96.66	**54.56**	67.71	86.95	*57.10*	60.93	60.34
	MSA	0.6380	0.1180	0.1565	0.1277	0.1144	0.0678	0.0775	0.0907	**0.0623**	0.0636	*0.0630*
	SAM	36.55	6.76	8.97	7.31	8.79	**3.42**	3.81	5.20	*3.57*	3.64	3.66
	Time (s)		654	618	194	*38*	1026	151	333	**13**	778	102
CASE3	MPSNR	15.50	27.63	24.29	30.98	29.58	33.49	30.11	30.50	33.37	*33.83*	**34.10**
	MSSIM	0.2364	0.7313	0.5596	0.8879	0.9065	0.9348	0.8870	0.8542	0.9349	*0.9564*	**0.9601**
	ERGAS	703.66	277.50	250.67	105.90	180.37	134.88	154.29	113.28	129.39	*69.36*	**66.17**
	MSA	0.6996	0.3503	0.2551	0.1380	0.2438	0.1787	0.1934	0.0996	0.1743	*0.0707*	**0.0666**
	SAM	40.09	20.07	14.61	7.90	11.05	10.24	11.08	5.70	9.99	**3.93**	*5.32*
	Time (s)		663	662	198	*38*	1123	149	351	**15**	1212	103
CASE4	MPSNR	12.05	14.85	15.44	15.69	15.57	15.55	15.56	**15.81**	15.52	15.74	*15.78*
	MSSIM	0.1788	0.4165	0.4690	0.6495	0.6940	0.6884	0.6942	0.6301	0.6657	*0.7347*	**0.7382**
	ERGAS	972.76	702.21	653.25	635.87	642.77	647.47	647.86	**625.63**	650.13	632.47	*632.01*
	MSA	0.5333	0.3172	0.2778	0.2659	0.2977	0.2526	0.2521	0.2439	0.2562	*0.2366*	**0.2360**
	SAM	30.55	18.18	15.92	15.24	17.59	14.48	14.33	13.97	14.68	**13.48**	*13.94*
	Time (s)		673	625	196	*37*	1179	161	362	**15**	1129	107

**Table 2:** Comparison of denoising metrics of different algorithms on the Pavia C dataset.

Pavia C Dataset												
Noise Case	Index	Noisy Data	BM4D	LRTV	LRMR	FastHyDe	GLF	NGmeet	LRTDTV	FastHyMix	SNLRSF	SNLTAI
CASE1	MPSNR	13.98	29.77	24.35	27.95	31.84	33.76	*34.43*	29.96	33.49	33.76	**34.67**
	MSSIM	0.1734	0.8423	0.5910	0.7996	0.9274	0.9394	*0.9475*	0.8495	0.9352	0.9373	**0.9504**
	ERGAS	666.14	107.37	200.45	133.37	86.28	68.15	*62.98*	106.30	70.60	68.17	**61.26**
	MSA	0.7287	0.1080	0.2477	0.1604	0.0878	0.0601	*0.0516*	0.1319	0.0643	0.0592	**0.0474**
	SAM	41.75	6.19	14.19	9.19	4.11	3.48	*2.80*	7.56	3.68	3.37	**2.73**
	Time (s)		156	222	61	*21*	416	90	109	**6**	517	39
CASE2	MPSNR	16.61	31.32	25.59	29.85	33.75	*35.67*	32.97	32.20	35.15	35.66	**36.52**
	MSSIM	0.2730	0.8836	0.6580	0.8550	0.9452	*0.9593*	0.8963	0.8883	0.9536	0.9580	**0.9601**
	ERGAS	512.32	90.12	186.27	109.48	69.61	**54.86**	108.84	95.79	58.68	55.60	*55.35*
	MSA	0.6118	0.0927	0.2309	0.1376	0.0703	*0.0511*	0.1377	0.1159	0.0564	0.0515	**0.0510**
	SAM	35.05	5.31	13.23	7.88	3.54	2.97	6.82	6.64	3.23	*2.95*	**2.72**
	Time (s)		150	227	65	*22*	416	77	108	**7**	612	39
CASE3	MPSNR	8.42	10.10	11.53	25.09	14.83	17.98	17.63	**27.17**	18.09	19.01	*26.50*
	MSSIM	0.0403	0.0546	0.0721	0.6820	0.6529	0.5866	0.4785	**0.7827**	0.6180	0.6276	*0.7788*
	ERGAS	1268.98	1047.52	891.19	185.26	638.07	428.61	456.35	*158.97*	425.77	383.12	**157.92**
	MSA	0.8110	0.7076	0.6144	0.1737	0.1719	0.1370	0.1838	0.1326	0.1375	*0.1235*	**0.1069**
	SAM	46.47	40.54	35.20	9.95	10.88	7.84	9.69	7.59	7.88	*6.92*	**6.13**
	Time (s)		142	223	62	*21*	717	77	124	**9**	670	35
CASE4	MPSNR	6.23	7.18	7.99	12.76	9.18	10.07	10.00	**13.48**	10.00	10.27	*13.13*
	MSSIM	0.0298	0.0392	0.0540	0.4690	0.3769	0.4360	0.3628	**0.5709**	0.3506	0.4766	*0.5686*
	ERGAS	1637.38	1469.89	1340.42	769.22	1203.47	1057.30	1067.07	*797.07*	1065.81	1016.81	**736.68**
	MSA	0.6220	0.5394	0.4619	0.1744	0.2116	0.1591	0.1767	0.1410	0.1796	**0.1270**	*0.1359*
	SAM	35.64	30.90	26.46	10.00	11.51	9.21	11.77	*7.50*	10.29	**7.31**	7.79
	Time (s)		147	223	62	55	728	117	124	**9**	679	*35*

## Data Availability

Data are contained within the article.
